# The compatibility of new blood pressure reference values for Korean children and adolescents with the US reference: the Korean Working Group of Pediatric Hypertension

**DOI:** 10.1186/s40885-022-00200-x

**Published:** 2022-08-01

**Authors:** Sung Hye Kim, Young Hwan Song, Hyo Soon An, Jae Il Shin, Jin-Hee Oh, Jung Won Lee, Seong Heon Kim, Hae Soon Kim, Hye-Jung Shin, Il-Soo Ha

**Affiliations:** 1 Department of Pediatrics, CHA Bundang Medical Center, CHA University, Seongnam, Republic of Korea; 2 The Korean Working Group of Pediatric Hypertension, Seoul, Republic of Korea; 3 Department of Pediatrics, Seoul National University Bundang Hospital, 82, Gumi-ro 173 Beon-gil, Bundang-gu, 13620 Seongnam, Republic of Korea; 4 Department of Pediatrics, Seoul National University Children’s Hospital, Seoul, Republic of Korea; 5 Department of Pediatrics, Yonsei University College of Medicine, Seoul, Republic of Korea; 6 Department of Pediatrics, The Catholic University of Korea, St. Vincent’s Hospital, Suwon, Republic of Korea; 7 Department of Pediatrics, Ewha Womans University College of Medicine, Seoul, Republic of Korea; 8 Department of Pediatrics, National Medical Center, Seoul, Republic of Korea

**Keywords:** Blood pressure, Hypertension, Adolescent

## Abstract

**Background:**

Childhood hypertension is associated with hypertension and metabolic syndrome in adulthood. Since the definition of childhood hypertension is based on the distribution of normative blood pressure (BP), a reference range is essential to create hypertension guidelines for children. We aimed to investigate the compatibility of the new Korean BP reference with the United States (US) BP reference based on the 2017 Clinical Practice Guideline.

**Methods:**

We compared the new Korean reference BP values for children and adolescents aged 10 to 17 years with those in the 2017 Clinical Practice Guidelines. We also analyzed the differences in the prevalence of hypertension in Korean children and adolescents when reference value was applied. Considering Korean and US BP references together, linear trend lines were sought.

**Results:**

Systolic BP (SBP) and diastolic BP (DBP) values in 95th percentiles showed no significant differences between the two BP references. Applying the two reference values, there was no significant difference in the prevalence of elevated BP and a combination of elevated BP and hypertension. Combining the Korean and US BP values and plotting them against age, approximate lines for the 90th and 95th SBP and DBP percentiles were observed.

**Conclusions:**

The BP values of the new Korean BP reference were similar to those of the US BP reference; they were reliable and interchangeable.

**Supplementary Information:**

The online version contains supplementary material available at 10.1186/s40885-022-00200-x.

## Background

Diagnosing hypertension in childhood is necessary because it significantly correlates with hypertension and metabolic syndrome later in life [1–3]. The definition of hypertension in adults is based on the risk of cardiovascular disease, and the cutoff values are universally applied, regardless of ethnicity or race [4, 5]. However, the definition of hypertension in childhood is based on the normative distribution of blood pressure (BP) in the pediatric population [6, 7], making an accurate definition of the reference range crucial for diagnosing hypertension in children. Worldwide, the most commonly used guidelines are the American or European guidelines, in which hypertension is defined as systolic BP (SBP) and/or diastolic BP (DBP) equal to greater than the 95th percentile. The most widely used reference values for BP were provided by The Fourth Report on the Diagnosis, Evaluation and Treatment of High Blood Pressure in Children and Adolescents by the National High Blood Pressure Education Program Working Group on High Blood Pressure in Children and Adolescents in 2004, which considered the data from several studies predominantly conducted in the United States (US), including the National Health and Nutrition Examination Survey (NHANES) [8]. Subsequently, in the Clinical Practice Guideline for Screening and Management of High Blood Pressure in Children and Aadolescents by the American Academy of Pediatrics published in 2017, BP reference values were revised by excluding the data of children and adolescents who were overweight and obese to minimize the effects of BP in this population on normative BP values [7]. The hypertension guidelines for children and adolescents in Europe and Canada continue to follow the BP reference values mentioned in The Fourth Report [6, 9].

However, there have been concerns that BP might differ according to ethnicity or race [10, 11], and several countries have established nation-specific BP references according to the age, sex, and height of their population for specific age groups [12, 13]. The Korean Working Group of Pediatric Hypertension also published the Korean BP reference values based on the data of children and adolescents aged 10 to 18 years with normal weight for their age [14]. However, since most guidelines on hypertension are based on the US BP reference values, it might be inappropriate to use native BP references for diagnosing hypertension if there is a wide difference between these values and those of the US.

Therefore, we aimed to investigate the difference between the new Korean BP reference and the reference indicated in the 2017 Clinical Practice Guideline and assess the possible correlation between BP values and age.

## Methods

### Comparison of the blood pressure reference values

We evaluated the differences in the reference values for SBP and DBP between the new Korean reference and the 2017 Clinical Practice Guideline for children and adolescents aged 10 to 17 years. Total SBP and DBP values were compared, as was each BP values of the 50th, 90th, and 95th percentiles for the different age and height percentile groups.

The new Korean BP reference values (Korean BP reference) were established based on the data from the Korean NHANES (KNHANES) from 1998 to 2016. The data comprised BP values of 10,442 participants (5,489 boys and 4,953 girls) aged 10 to 18 years who had a normal weight according to their sex and age. BP was measured using a mercury sphygmomanometer in the standard manner as suggested by the guidelines [7]. The BP reference values contained sex-, age-, and height-specific SBP and DBP values.

The BP reference values in the 2017 Clinical Practice Guideline (the US BP reference) are also based on auscultatory BP measurements of 49,967 children and adolescents aged 1 to 17 years with normal weight for their age [15]. These data were retrieved from several studies from 1976 to 2000, including the NHANES. The US BP reference values also contained sex-, age-, and height-specific SBP and DBP values.

### Comparison of the prevalence of hypertension according to the reference values

To compare the Korean BP reference to the US BP reference in terms of hypertension prevalence, we categorized elevated BP and hypertension using the BP data of children and adolescents aged 10 to 17 years from the KNHANES database from 2007 to 2018. Elevated BP was defined as BP between the range of ≥120/80 mmHg and <95th percentile, or ≥90th and <95th percentile, whichever was lower, and hypertension was defined as SBP and/or DBP ≥95th percentile [7].

This study was approved by the Institutional Review Board of the Bundang CHA Medical Center (No. 2021-03-025). The requirement for informed consent was waived due to the retrospective nature of the study.

### Modified simple BP classification

From the Korean and US BP reference ranges, we combined the SBP and DBP values of the 90th and 95th percentiles at the 50th percentile height in different age groups and estimated the approximate BP values comparable to these BP reference values according to age. By using these approximate BP values, we established the modified simple BP classification.

### Statistical analyses

The variables were expressed as means with standard deviation; differences in these variables were evaluated using the Mann-Whitney test. The McNemar test was used to compare the prevalence of hypertension calculated using the new Korean BP reference and the US BP reference.

The approximate BP values using the modified simple BP classification were estimated; using the paired t-test, these were compared with the BP values at the 50th percentile height in different age groups to evaluate the similarities between the two. Analyses were conducted using IBM SPSS ver. 18.0 (IBM Corp., Armonk, NY, USA). Statistical significance was defined as *P* < 0.05.

## Results

### Comparison of the BP reference values

**Fig. 1:**
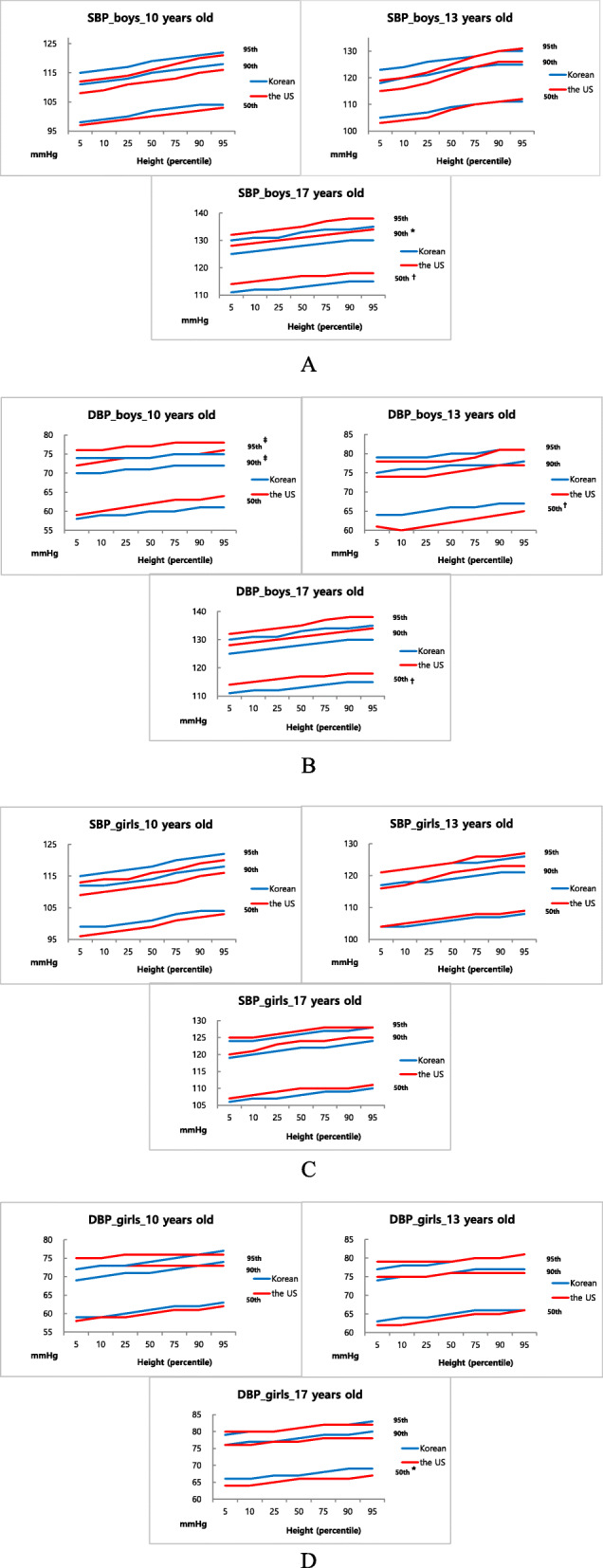
Comparison of the 50th, 90th, and 95th percentile blood pressure values between Korean and American children and adolescents. The ages in the graphs have been selected randomly. **A** Systolic blood pressure (SBP) in boys. **B** Diastolic blood pressure (DBP) in boys. **C** SBP in girls. **D** DBP in girls. ^*^0.01 ≤ *P* < 0.05, ^†^0.005 ≤ *P* < 0.01, ^‡^*P* < 0.005

Overall, the Korean BP reference values were similar to the US BP reference values without a significant statistical difference (Table 1). Comparing BP values between the two BP references in each age group, the 95th percentiles of SBP and DBP showed no statistical differences, except for DBP in ages 10 and 11 (Table 2). SBP values in Korean children aged 10 years were slightly higher than those in American children for both sexes; however, the difference was not statistically significant. In contrast, SBP values for the 95th percentile in Korean adolescents aged 17 years were slightly lower than those of the US adolescents but without statistical significance (Fig. 1). Table 2 shows the data of differences between the Korean and US BP reference values for different age groups and their statistical significance.

**Table 1:** Difference between the Korean and US BP reference values

Blood press	Difference (mmHg)	
	Boy	Girl
Systolic BP	0.3 ± 2.0 (–4 to 4)	0.4 ± 1.5 (–2 to 4)
Diastolic BP	0.6 ± 2.1 (–3 to 5)	0.3 ± 1.6 (–5 to 3)

Data are presented as mean ± standard deviation (range). All P-values >0.05

BP, blood pressure

**Table 2:** Difference between the Korean and US BP reference values in different age groups

Variable	Age (yr)	Boy, difference (mmHg)			Girl, difference (mmHg)		
		BP percentile 50th	BP percentile 90th	BP percentile 95th	BP percentile 50th	BP percentile 90th	BP percentile 95th
Systolic BP							
	10	1.4 ± 0.2 (1 to 2)	2.5 ± 0.5 (2 to 3)	2.4 ± 0.9 (1 to 3)	2.1 ± 0.6 (1 to 3)	2.2 ± 0.4 (2 to 3)	2.3 ± 0.6 (2 to 3)
	11	1.9 ± 0.5 (1 to 3)	3.2 ± 0.8 (3 to 4)	3.1 ± 1.2 (1 to 5)	1.3 ± 1.1 (0 to 3)	1.7 ± 1.2 (–1 to 3)	1.7 ± 1.4 (0 to 3)
	12	1.8 ± 0.9 (0 to 3)	2.6 ± 0.7 (1 to 3)	2.9 ± 1.8 (0 to 5)	–0.3 ± 1.0 (–2 to 1)	0.0 ± 1.5 (–2 to 1)	0.2 ± 1.2 (–1 to 1)
	13	0.8 ± 1.3 (–1 to 2)	1.4 ± 2.0 (–1 to 4)	1.8 ± 2.1 (–1 to 4)	–0.8 ± 0.4 (–1 to 0)	–0.8 ± 1.2 (–2 to 1)	–0.7 ± 0.6 (–2 to 0)
	14	0.4 ± 1.5 (–1 to 3)	–0.1 ± 1.9 (–2 to 2)	–0.2 ± 2.1 (–2 to 3)	–0.9 ± 0.5 (–2 to 0)	–0.9 ± 1.0 (–2 to 1)	–0.8 ± 0.3 (–1 to 0)
	15	–0.5 ± 1.0 (–1 to 1)	–1.1 ± 1.1 (–2 to 1)	–1.2 ± 1.2 (–2 to 1)	–0.3 ± 0.5 (–1 to 1)	–0.7 ± –0.9 (–1 to 1)	–1.0 ± 0.3 (–1 to –1)
	16	–1.6 ± 0.7 (–3 to –1)	–2.1 ± 0.6 (–3 to –1)	–2.1 ± 0.8 (–3 to –1)	–0.8 ± 0.4 (–1 to 0)	–1.2 ± 0.8 (–2 to 0)	–1.0 ± 0.4 (–2 to –1)
	17	–3.2 ± 0.4 (–4 to –3)^b^	–3.2 ± 0.4 (–4 to –3)^a^	–2.8 ± 0.6 (–4 to –2)	–1.2 ± 1.2 (–2 to –1)	–1.6 ± 1.5 (–2 to –1)	–1.0 ± 1.4 (–1 to 0)
Diastolic BP							
	10	–2.0 ± 0.7 (–3 to –1)	–3.0 ± 0.6 (–4 to –2)^c^	–2.7 ± 0.3 (–3 to –2)^c^	0.8 ± 0.3 (0 to 1)	–1.4 ± 1.5 (–3 to 1)	–1.3 ± 1.4 (–3 to 1)
	11	–0.6 ± 0.6 (–1 to 0)	–2.1 ± 0.3 (–3 to –2)^c^	–1.6 ± 0.6 (–3 to –1)^c^	1.1 ± 0.5 (0 to 2)	–1.0 ± 1.3 (–3 to 0)	–0.5 ± 1.6 (–2 to 2)
	12	1.5 ± 0.9 (0 to 2)	–0.5 ± 0.7 (–1 to 0)	–0.3 ± 0.6 (–1 to 0)	1.2 ± 0.8 (0 to 2)	–0.7 ± 1.1 (–2 to 1)	–0.6 ± 1.1 (–2 to 1)
	13	3.2 ± 0.7 (2 to 4)^b^	1.2 ± 0.6 (0 to 2)	0.6 ± 0.8 (0 to 2)	1.2 ± 0.5 (0 to 2)	0.2 ± 0.9 (–1 to 1)	–0.6 ± 0.8 (–2 to 0)
	14	3.8 ± 1.7 (2 to 6)^b^	1.5 ± 1.7 (–1 to 3)	0.8 ± 2.0 (–2 to 3)	1.1 ± 0.5 (0 to 2)	0.1 ± 0.8 (–1 to 1)	–0.9 ± 0.7 (–2 to 0)
	15	4.0 ± 1.5 (2 to 6)^c^	1.1 ± 1.7 (–1 to 4)	0.7 ± 2.3 (–2 to 4)	1.2 ± 0.4 (1 to 2)	0.3 ± 0.4 (0 to 1)	–0.7 ± 0.4 (–1 to 0)
	16	3.3 ± 1.4 (2 to 6)^b^	1.0 ± 1.3 (0 to 3)	0.5 ± 1.8 (–1 to 3)	1.4 ± 0.3 (1 to 2)	0.5 ± 0.5 (0 to 1)	–0.3 ± 0.5 (–1 to 0)
	17	3.1 ± 0.9 (2 to 5)^b^	1.4 ± 1.1 (0 to 3)	0.9 ± 1.7 (–1 to 4)	2.0 ± 0.4 (1 to 3)^a^	0.8 ± 0.5 (0 to 2)	0.1 ± 0.5 (–1 to 1)

Data are presented as mean ± standard deviation (range)

BP, blood pressure

^a^0.01 ≤ *P* < 0.05, ^b^0.005 ≤ *P* < 0.01, ^c^*P* < 0.005

Similarly, comparing BP values in different height groups, the 95th percentiles of SBP and DBP were not significantly different (Table 3). Comparing BP values in different height percentile groups showed that SBP values of the 5th and 10th height percentiles were higher in Korean children, but those of the 75th to 95th percentiles were higher in American children without statistical significance (Table 3).

**Table 3:** Difference between the Korean and US BP reference values in different height percentile groups

Variable	Height percentile	Boy, difference (mmHg)			Girl, difference (mmHg)		
		BP percentile 50th	BP percentile 90th	BP percentile 95th	BP percentile 50th	BP percentile 90th	BP percentile 95th
Systolic BP							
	5th	1.0 ± 1.9 (–3 to 3)	1.6 ± 2.5 (–3 to 4)	2.1 ± 2.4 (–2 to 4)	0.7 ± 1.4 (–1 to 3)	1.0 ± 1.2 (–1 to 3)	0.3 ± 1.7 (–1 to 3)
	10th	0.8 ± 2.1 (–3 to 3)	1.3 ± 2.6 (–3 to 4)	1.7 ± 2.7 (–2 to 5)	0.3 ± 1.5 (–1 to 2)	0.7 ± 1.2 (–1 to 2)	0.3 ± 1.4 (–1 to 2)
	25th	0.2 ± 2.3 (–4 to 3)	0.9 ± 2.7 (–3 to 4)	1.3 ± 3.0 (–3 to 5)	–0.1 ± 1.4 (–2 to 2)	0.0 ± 1.8 (–2 to 2)	0.4 ± 1.7 (–1 to 3)
	50th	–0.2 ± 2.2 (–4 to 2)	0.2 ± 2.6 (–3 to 3)	0.6 ± 2.6 (–2 to 3)	–0.3 ± 1.5 (–2 to 2)	–0.5 ± 2.0 (–2 to 3)	0.0 ± 1.7 (–2 to 3)
	75th	–0.2 ± 1.8 (–3 to 2)	–0.2 ± 2.6 (–3 to 3)	–0.3 ± 2.5 (–3 to 3)	–0.6 ± 1.1 (–2 to 2)	–0.7 ± 1.9 (–2 to 3)	–0.3 ± 1.7 (–2 to 3)
	90th	–0.3 ± 1.8 (–3 to 2)	–0.4 ± 2.3 (–3 to 3)	–0.9 ± 2.0 (–4 to 1)	–0.4 ± 1.0 (–2 to 2)	–0.8 ± 1.4 (–2 to 2)	–0.4 ± 1.0 (–1 to 2)
	95th	–0.4 ± 1.3 (–3 to 1)	–1.0 ± 3.1 (–7 to 3)	–1.1 ± 1.7 (–3 to 1)	–0.4 ± 0.8 (–1 to 1)	–0.9 ± 1.0 (–2 to 2)	–0.6 ± 1.0 (–1 to 2)
Diastolic BP							
	5th	3.0 ± 3.0 (–1 to 6)	1.1 ± 2.5 (–2 to 4)	1.2 ± 2.7 (–2 to 4)	1.3 ± 0.4 (1 to 2)	–1.3 ± 1.2 (–3 to 0)	–1.7 ± 0.7 (–3 to –1)
	10th	2.8 ± 3.0 (–1 to 6)	0.8 ± 2.4 (–3 to 3)	0.8 ± 2.4 (–3 to 3)	1.5 ± 0.6 (0 to 2)	–1.0 ± 1.4 (–3 to 1)	–1.4 ± 0.8 (–2 to 0)
	25th	2.1 ± 2.5 (–2 to 5)	0.3 ± 2.3 (–3 to 3)	0.2 ± 1.8 (–3 to 2)	1.6 ± 0.4 (1 to 2)	–0.4 ± 1.2 (–3 to 1)	–0.8 ± 0.9 (–3 to 0)
	50th	1.8 ± 2.4 (–2 to 4)	0.0 ± 1.7 (–3 to 2)	–0.2 ± 1.4 (–3 to 2)	1.3 ± 0.5 (1 to 2)	–0.1 ± 0.8 (–2 to 1)	–0.4 ± 0.6 (–2 to 0)
	75th	1.6 ± 2.2 (–3 to 3)	–0.3 ± 1.5 (–3 to 1)	–0.7 ± 1.2 (–3 to 0)	1.0 ± 0.6 (0 to 2)	0.2 ± 0.5 (–1 to 1)	–0.4 ± 0.4 (–1 to 0)
	90th	1.4 ± 1.7 (–2 to 3)	–0.7 ± 1.3 (–3 to 1)	–1.0 ± 0.9 (–3 to 0)	1.1 ± 0.7 (0 to 3)	0.6 ± 0.5 (0 to 1)	0.3 ± 0.4 (0 to 1)
	95th	1.3 ± 1.8 (–3 to 2)	–0.8 ± 1.4 (–4 to 1)	–1.1 ± 0.9 (–3 to 0)	0.9 ± 0.5 (0 to 2)	1.0 ± 0.4 (0 to 2)	0.4 ± 0.7 (–1 to 2)

Data are presented as mean ± standard deviation (range). All P-values >0.05

BP, blood pressure

### Comparison of the prevalence of hypertension according to the reference values

A total of 9,095 children and adolescents aged 10 to 17 years who participated in the KNHANES from 2007 to 2018 were included in the hypertension prevalence analysis. Elevated BP was diagnosed in 8.3% and 8.0%, and hypertension was diagnosed in 9.5% and 9.9% of patients based on the Korean and US reference ranges, respectively (*P* = 0.120 and *P* < 0.001, respectively). A combination of elevated BP and hypertension was diagnosed in 17.8% and 17.9% of patients, respectively (*P* = 0.422).

### Correlation between BP values and age and the modified simple BP classification

**Fig. 2:**
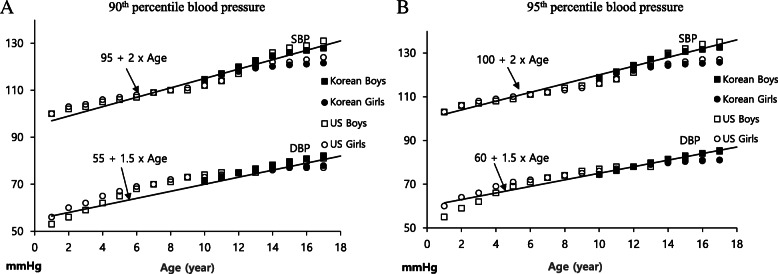
The (**A**) 90th and (**B**) 95th percentile blood pressure values at the 50th percentile height in the different age groups, with the approximate trend lines

By using age, we estimated the approximate BP values comparable to the SBP and DBP values for the 90th and 95th percentiles at the 50th percentile height in boys and girls in Korea and the US. When performing the paired t-test to evaluate the similarity between these two values, the estimated BP values using age and the percentile BP values had very strong correlations each other (all correlation coefficients >0.95, all *P* < 0.001), and had no statistically significant differences (all mean paired differences <1 mmHg; 95% confidence interval of difference, –1.5 to 1.5 mmHg; all P-values for difference, >0.05) (Table 4). We established the modified simple BP classification for children and adolescents, using the estimated BP values using age (Table 5). Figure 2 shows the trend lines of the SBP and DBP values for the 90th and 95th percentiles at the 50th percentile height and the estimated BP value lines.

**Table 4:** Comparison between the estimated approximate BP values and the percentile BP values at the 50th percentile height

Variable	Correlation coefficient	P-value for correlation	Paired difference		
			Mean difference (mmHg)	95% Confidence interval of difference (mmHg)	P-value for difference
SBP 95th P vs. (age × 2) + 99	0.961	<0.001	–0.712	–1.480 to 0.057	0.069
SBP 90th P vs. (age × 2) + 95	0.962	<0.001	–0.558	–1.346 to 0.231	0.162
DBP 95th P vs. (age × 1.5) + 60	0.955	<0.001	–0.269	–0.878 to 0.339	0.379
DBP 90th P vs. (age × 1.5) + 56	0.953	<0.001	0.500	–0.124 to 1.124	0.114

BP, blood pressure; SBP, systolic BP; P, percentile; DBP, diastolic BP

**Table 5:** Modified simple BP classification in children and adolescents

Category	1–17 years		≥18 years
	SBP and/or DBP percentile	(SBP – 2 × age) and/or (DBP – 1.5 × age) (mmHg)	SBP and/or DBP (mmHg)
Normal	<90th	<95 / <55	<130 / <85
Prehypertension	≥ 90th to < 95th	95–99 / 55–59	130–139 / 85–89
Stage 1 hypertension	≥95th to <99th + 5 mmHg	100–114 / 60–69	140–159 / 90–99
Stage 2 hypertension	≥99th + 5 mmHg	≥115 / ≥70	≥160 / ≥100

BP, blood pressure; SBP, systolic BP; DBP, diastolic BP

## Discussion

Our study showed that the new Korean BP reference values and US BP reference are similar. Applying the two BP reference ranges to Korean children and adolescents, the prevalence of elevated BP and a combination of elevated BP and hypertension were not significantly different between the two ranges.

Since the definition of hypertension is based on the normative distribution of BP, the reference values for BP are crucial. There are several issues related to establishing BP reference values. Until now, major guidelines have recommended auscultatory BP measurement as the standard method [6–8], and the new Korean BP reference range was also established based on BP values recorded using the same measurement method. Another issue is the effect of excess weight and obesity on normative BP values. The recently published BP references, including the new Korean BP references, exclusively included the BP data of children and adolescents with normal weight [7, 13, 14, 16].

Another challenge related to the BP reference range is the ethnic differences in BP values. In a study comparing BP differences between blacks and whites among children and adolescents in the US, the differences were small and inconsistent [17]. In contrast, in a study of BP among British adolescents, SBP in the minority ethnic groups was generally lower than that in the major ethnic group [10], and the change in BP was greater in black Africans than in whites in late adolescence, revealing the effect of ethnicity on BP in adolescents [11]. Racial/ethnic differences in BP were reflected in the study by Hardy et al. [18]. They reported disparities in the prevalence of ideal BP, prehypertension, and hypertension during the course of life among African, white, and Mexican adolescents. Hence, several groups have published BP references for the native population based on their data of children and adolescents [12, 13, 16, 19]. Similarly, the Korean Working Group of Pediatric Hypertension has established the new Korean BP reference values [14].

BP reference values for Koreans have been published previously. One of them was derived from the data of oscillometric BP references using Dinamap ProCare 200 (GE Inc., Milwaukee, WI, USA) in children and adolescents aged 7 to 20 years by Lee et al. [20]. The data of children and adolescents with weights exceeding the average by three standard deviations in the same age and height percentiles were excluded. In these BP reference data, the SBP and DBP values for the 95th percentile in 10-year-old boys were 8 to 9 mmHg and 4 to 5 mmHg higher, respectively, than the US BP reference values based on auscultatory BP measurements. In a validation study, this device failed to meet the standards set by the 2010 International Protocol of the European Society of Hypertension, and the mean difference in the absolute BP values measured with this device and the mercury sphygmomanometer was 1.85 ± 1.65 mmHg for SBP and 4.41 ± 3.53 mmHg for DBP [21]. Compared with Lee et al. [21]’s BP reference values, the new Korean BP reference did not show a significant difference in 10-year-old boys (95th percentile SBP, 1 to 3 mmHg and 95th percentile DBP, –3 to –2 mmHg) compared with the US reference. Another reference was established based on the data of auscultatory BP measurements; however, it comprises the data of children and adolescents who were overweight and obese [22].

In this study, the Korean reference SBP values were slightly higher for 10-year-olds, but lower for 17-year-olds than the corresponding US reference values. Each BP reference table also provides cutoff values of height percentile in each age group. Korean cutoff values of height percentiles in 10-year-old boys were slightly higher than those of the US (Korean BP reference vs. US BP reference, 0.3 ± 1.0 cm) and vice versa in 17-year-old boys (–2.6 ± 2.1 cm), which could affect the BP values since height is an important factor affecting BP. However, DBP did not show a similar trend, suggesting that height could have had more effect on SBP than DBP.

According to the BP reference values, the diagnosis of hypertension can vary, leading to confusion. Dong et al. [12] published a new Chinese BP reference range and analyzed the prevalence of elevated BP. The prevalence of elevated BP among children aged 7 to 17 years based on the new Chinese reference was between 7.8 and 18.5% compared with 4.3–14.5% based on the BP reference from The Fourth Report. In this Chinese BP reference, the 95th and 99th percentiles BP values were higher for boys aged 7 to 15 years and lower for boys aged 16 to 17 years than those from The Fourth Report [12]. However, applying the new Korean BP reference, elevated BP prevalence was similar to that of the US reference in our study.

The new Korean reference values were established using auscultatory BP data from KNHANES, a well-designed and controlled national survey conducted by the Korean Centers for Disease Control and Prevention. It showed no significant difference compared to US BP reference values; thus, the prevalence of hypertension based on both BP reference ranges was similar, making this new Korean BP reference more useful and reliable.

The recognition of hypertension in childhood is lower than the actual prevalence since the diagnosis of hypertension in this age group is complicated [23]. Hence, we created a simplified formula to diagnose hypertension. SBP and DBP values based on the new Korean BP reference and the US BP references showed good correlation with age, as observed from the trend lines. Thus, we could establish a modified simple BP classification method for children and adolescents.

This study had some limitations. First, we could compare the BP values only from both the BP reference tables and not the raw data; this could have led to selection bias. Second, the analysis was conducted for subjects aged 10 to 17 years since the new Korean BP reference does not provide a BP reference range for the lower age groups. The Korean Working Groups for Pediatric Hypertension are working on this.

## Conclusions

The new Korean BP reference was established using the data of Korean children and adolescents with normal weight for their age, and the values were similar to those of the US BP reference and were reliable and interchangeable. We also created simple formulas to detect hypertension easily and provided a modified simple BP classification.

## Supplementary information

**Additional file 1**

## Data Availability

All data generated or analyzed during this study are included in previously published articles [7, 14].

## Abbreviations

BP: Blood pressure
DBP: Diastolic blood pressure
KNHANES: Korean National Health and Nutrition Examination Survey
NHANES: National Health and Nutrition Examination Survey
SBP: Systolic blood pressure
US: United States
